# NKG2A-Expressing Natural Killer Cells Dominate the Response to Autologous Lymphoblastoid Cells Infected with Epstein–Barr Virus

**DOI:** 10.3389/fimmu.2016.00607

**Published:** 2016-12-15

**Authors:** Olivia Hatton, Dara Marie Strauss-Albee, Nancy Q. Zhao, Mikel D. Haggadone, Judith Shanika Pelpola, Sheri M. Krams, Olivia M. Martinez, Catherine A. Blish

**Affiliations:** ^1^Department of Molecular Biology, Colorado College, Colorado Springs, CO, USA; ^2^Program in Immunology, Stanford University School of Medicine, Stanford, CA, USA; ^3^Department of Surgery, Stanford University School of Medicine, Stanford, CA, USA; ^4^Department of Medicine, Stanford University School of Medicine, Stanford, CA, USA

**Keywords:** Epstein–Barr virus, NKG2A, NK cells, B cells, lymphoblastoid cell lines

## Abstract

Epstein–Barr virus (EBV) is a human γ-herpesvirus that establishes latency and lifelong infection in host B cells while achieving a balance with the host immune response. When the immune system is perturbed through immunosuppression or immunodeficiency, however, these latently infected B cells can give rise to aggressive B cell lymphomas. Natural killer (NK) cells are regarded as critical in the early immune response to viral infection, but their role in controlling expansion of infected B cells is not understood. Here, we report that NK cells from healthy human donors display increased killing of autologous B lymphoblastoid cell lines (LCLs) harboring latent EBV compared to primary B cells. Coculture of NK cells with autologous EBV^+^ LCL identifies an NK cell population that produces IFNγ and mobilizes the cytotoxic granule protein CD107a. Multi-parameter flow cytometry and Boolean analysis reveal that these functional cells are enriched for expression of the NK cell receptor NKG2A. Further, NKG2A^+^ NK cells more efficiently lyse autologous LCL than do NKG2A^−^ NK cells. More specifically, NKG2A^+^2B4^+^CD16^−^CD57^−^NKG2C^−^NKG2D^+^ cells constitute the predominant NK cell population that responds to latently infected autologous EBV^+^ B cells. Thus, a subset of NK cells is enhanced for the ability to recognize and eliminate autologous, EBV-infected transformed cells, laying the groundwork for harnessing this subset for therapeutic use in EBV^+^ malignancies.

## Introduction

Epstein–Barr virus (EBV) is a ubiquitous γ-herpesvirus that persists as a chronic, asymptomatic infection in over 90% of the adult human population (1). EBV preferentially establishes infection in naïve tonsillar B cells. EBV remains in the lytic phase of infection in a minority of cells, producing infectious viral particles that are spread by oral transmission. Latent infection is established in the vast majority of infected cells and is responsible for driving infected B cells into the memory B cell reservoir, where the virus persists for the lifetime of the host; in healthy carriers, an average of 10 of every million peripheral blood memory B cells are EBV-infected (1, 2). Distinct viral proteins from both lytic and latent cycles of infection elicit a robust cellular immune response in the host.

While the T cell response to EBV-infected cells has been extensively studied (3), several lines of evidence from murine models and human studies suggest that natural killer (NK) cells are also critical in host immunity to EBV. First, in humanized murine models, depletion of NK cells increases features typical of infectious mononucleosis (IM) (4) and promotes EBV-associated tumorigenesis (4, 5). Second, human NK cells kill lytically infected allogeneic B cells *in vitro* (6, 7). Third, NK cell numbers expand during primary symptomatic EBV infection in IM patients (8, 9). Finally, patients with X-linked lymphoproliferative syndrome and X-linked immunodeficiency with Mg^2+^ defect, EBV infection, and neoplasia (XMEN) have NK deficiencies and suffer from life-threatening complications of EBV infection including IM and spontaneous EBV-associated malignancies (10–18). Notably, these complications appear to be related to NK cell function because they often occur in the presence of normal CD8^+^ T cell responses and involve defective NK receptor (NKR) expression or signaling (13–18).

Natural killer cells are phenotypically heterogeneous in their expression of inhibitory and activating NKRs (19). Inhibitory receptors include NKG2A and many of the killer immunoglobulin-like receptors (KIR), while activating NKRs include NKG2D, NKG2C, and the natural cytotoxicity receptors. Subsets of NK cells defined by their NKR expression have been described in response to specific pathogens. For example, NKG2C^+^ NK cells preferentially expand during acute human cytomegalovirus (CMV) infection as well as in CMV-seropositive individuals co-infected with hantavirus, chikungunya virus, chronic HIV, or chronic hepatitis B or C (20–26). Along similar lines, recent evidence suggests that particular NK cell subsets respond to EBV infection. For instance, a IFNγ^hi^CD56^bright^NKG2A^+^CD94^+^CD54^+^CD62L^−^ NK cell subset accumulates in the tonsils of EBV carriers and reduces B cell transformation by EBV more potently than other CD56^bright^ NK cells (27). Further, CD56^dim^KIR^−^NKG2A^+^ NK cells preferentially proliferate during acute EBV^+^ IM and degranulate in response to allogeneic B cells displaying EBV lytic antigens (7). Finally, a mature CD56^dim^NKG2A^+^CD57^+^ NK population persists after acute EBV infection in individuals co-infected with CMV (28).

Thus, various NKR and NK cell subsets have been implicated in the primary response to EBV-infected cells during acute IM and B cell transformation by EBV. However, latent infection dominates the landscape of EBV. Failure to control latent EBV infection can lead to serious disease, particularly from a variety of EBV-associated malignancies, including lymphoproliferative diseases (EBV-LPD). EBV-LPD represent a spectrum of potentially fatal lymphoproliferations, often involving B lymphocytes, which arise when the immune system is compromised by posttransplant immunosuppression, HIV, immunomodulating biologicals, or advancing age (29–32). The role of NK cells in the immune response to autologous cells latently infected with EBV is unclear. Thus, our goal was to assess the ability of NK cells to recognize and respond to autologous lymphoblastoid cell lines (LCLs), in order to better understand mechanisms that prevent expansion of latently infected cells in healthy individuals and to present new therapeutic opportunities for EBV-LPD.

## Materials and Methods

### LCL Generation, Primary B Cell and NK Cell Isolation, and Cell Lines

EBV^+^ LCLs were generated from 11 healthy donors by infection of freshly isolated PBMCs with the B95.8 laboratory strain of EBV, as previously described (33). LCL and the MHC-I^lo^ 721.221 cell line were maintained in RPMI (Corning) supplemented with 10% FBS (Serum Source International) and 1% penicillin/streptomycin (Corning) [complete RPMI (cRPMI)]. Primary NK cells or B cells were negatively selected from whole blood using the RosetteSep Human NK Enrichment Kit or Human B Cell Enrichment Kit, respectively (Stem Cell Technologies). Purity was routinely (90% (Figures S1A,B in Supplementary Material). Purified primary NK cells were cultured for 2 days in cRPMI supplemented with 300 U/mL IL-2 (NIH Reagent Program) prior to stimulation or coculture. This study was performed in accordance with the Declaration of Helsinki and approved by the Stanford University Institutional Review Board, and written informed consent was obtained from all participants.

### Cytotoxicity Assay

Natural killer cell cytotoxicity was assayed by a modified ACT1 assay (Cell Technology). Briefly, target cells (721.221, primary B cells, autologous LCL) were incubated with 0.25 µM CFSE in PBS + 2.5% FBS for 5 min at room temperature, then washed twice with 10 volumes cRPMI. A total of 0.5 × 10^5^ target cells were cocultured for 4 h in a 37°C-5% CO_2_ humidified incubator with 2 × 10^5^ NK cells, for a final ratio of 4 NK cells:1 target cell. Cocultures were pelleted, resuspended in 200 µL cRPMI, and incubated with 5 µL 7-aminoactinomycin D (7-AAD) for 15 min on ice. Unlabeled target cells served as a control for gating, while CFSE-labeled target cells treated with 1× final FACS Perm (BD Pharmingen) for the final 15 min of the coculture served as a positive control for maximum target cell death. Cells were analyzed on a FACScan flow cytometer with CellQuest Software, and dead target cells were identified as CFSE^+^7-AAD^+^. Specific killing was calculated as [(%CFSE^+^7-AAD^+^_NK:Target_ − % CFSE^+^7-AAD^+^_Target Only_)/(% CFSE^+^7-AAD^+^_Permeabilized Target_ − % CFSE^+^7-AAD^+^_Target Only_)] × 100.

For the cytotoxicity assay using purified NKG2A^+^ and NKG2A^−^ NK cells, NK cells were isolated using an NK Isolation Kit (Miltenyi) and cultured in RPMI complete with 300 U/ml IL-2 for 2 days. NK cells were then stained with NKG2A-PE (Beckman Coulter, clone Z199), CD3-APC (Biolegend), CD56-PE Cy7 (Biolegend), CD16-PerCP Cy5.5 (Biolegend), and Fixable Viability Dye eFluor 780 (eBioscience) and sorted for live CD3^−^CD56^+^CD16^+^ and NKG2A^±^ on a BD FACSAria. Sorted cells were rested in RPMI complete with 300 U/ml IL-2 for 2 h at 37°C before the start of the killing assay. LCLs were labeled with 0.5 µM CellTrace Violet (Invitrogen) for 20 min at 37°C, then washed twice with at least 10 volumes of RPMI complete. LCL target cells (2.5 × 10^4^) were cocultured with 1 × 10^5^ sorted NKG2A^+^ or NKG2A^−^ NK cells for 4 h in a 37°C incubator for a 4:1 E:T ratio. At the end of the coculture, cells were stained for viability using Fixable Viability Dye eFluor 780 (eBioscience) at the manufacturer’s recommended concentration, for 10 min on ice. Cells were then washed twice with PBS before analysis as above.

### Analysis of NK Ligand Expression

PBMCs were isolated from whole blood by Ficoll-Paque (GE Healthcare). PBMC and LCL were stained with the following antibodies or matched isotype controls: anti-CD48-PE, anti-CD19-FITC, anti-CD19-PE, anti-HLA-E-PE, and anti-HLA-A,B,C-FITC (BD Pharmingen) and analyzed by flow cytometry. All antibodies were titrated on positive control cell lines; these lines were stained as a positive control in each experiment. Stain indexes were used to compare NK ligand expression on CD19^+^-gated primary B cells and autologous LCL (34). Stain indexes were calculated using the mean fluorescence intensities (MFI) as (MFI_ligand_ − MFI_isotype_)/(2 × SD_isotype_).

### NK:Target Cell Cocultures and Flow Cytometric Analysis

IL-2 primed NK cells (0.25 × 10^6^ cells/condition) were cocultured with equal amounts of 721.221, primary B cells, or autologous LCL in 200 µL final volume (Figure S2A in Supplementary Material). NK cells were left unstimulated (NK only) as a negative control or were stimulated with 1× PMA/Ionomycin Cell Stimulation Cocktail (eBioscience) as a positive control. All cells were cultured in cRPMI supplemented with 300 U/mL IL-2, 1× final brefeldin A/monensin protein transport inhibitor (eBioscience), and anti-CD107a-Pacific blue (BioLegend). After 4 h incubation in a 37 C-5% CO_2_ humidified incubator, 1.8 mM EDTA was added for 5 min to arrest stimulation. Cells were stained with the near IR Live/Dead stain (Life Technologies), washed, and then stained with anti-CD3-Alexa 700, anti-CD14-Alexa 700, anti-CD19-Alexa 700, anti-CD56-Brilliant Violet 605, anti-NKG2D-PE,Cy7, anti-2B4-PerCP,Cy5.5 (BioLegend), anti-CD16-V500 (BD Pharmingen), anti-NKG2C-PE (R&D Systems), and anti-NKG2A-FITC (Miltenyi). Cells were then fixed and permeabilized with FACS Lyse and FACS Perm II (BD Pharmingen), according to the manufacturer’s instructions. Cells were then stained with anti-IFNγ-Brilliant Violet 785. All antibodies were titrated on PBMC prior to use. Fluorescence minus one-stained PBMC were run for each experiment and used to set gates for each parameter. Data were collected on a four-laser LSRII with FACS DiVA software at the Stanford Shared FACS Facility.

### Data and Statistical Analysis

Flow cytometry data were analyzed using FlowJo, version 9.7.6 (Tree Star). Statistical analysis was performed using Prism, version 6.0d (GraphPad Software). All paired data were compared using the Wilcoxon matched-pairs signed rank test. A Bonferroni correction was used to adjust for multiple comparisons.

## Results

### Expression of NK Cell Receptor Ligands on EBV^+^ LCL

**Figure 1:**
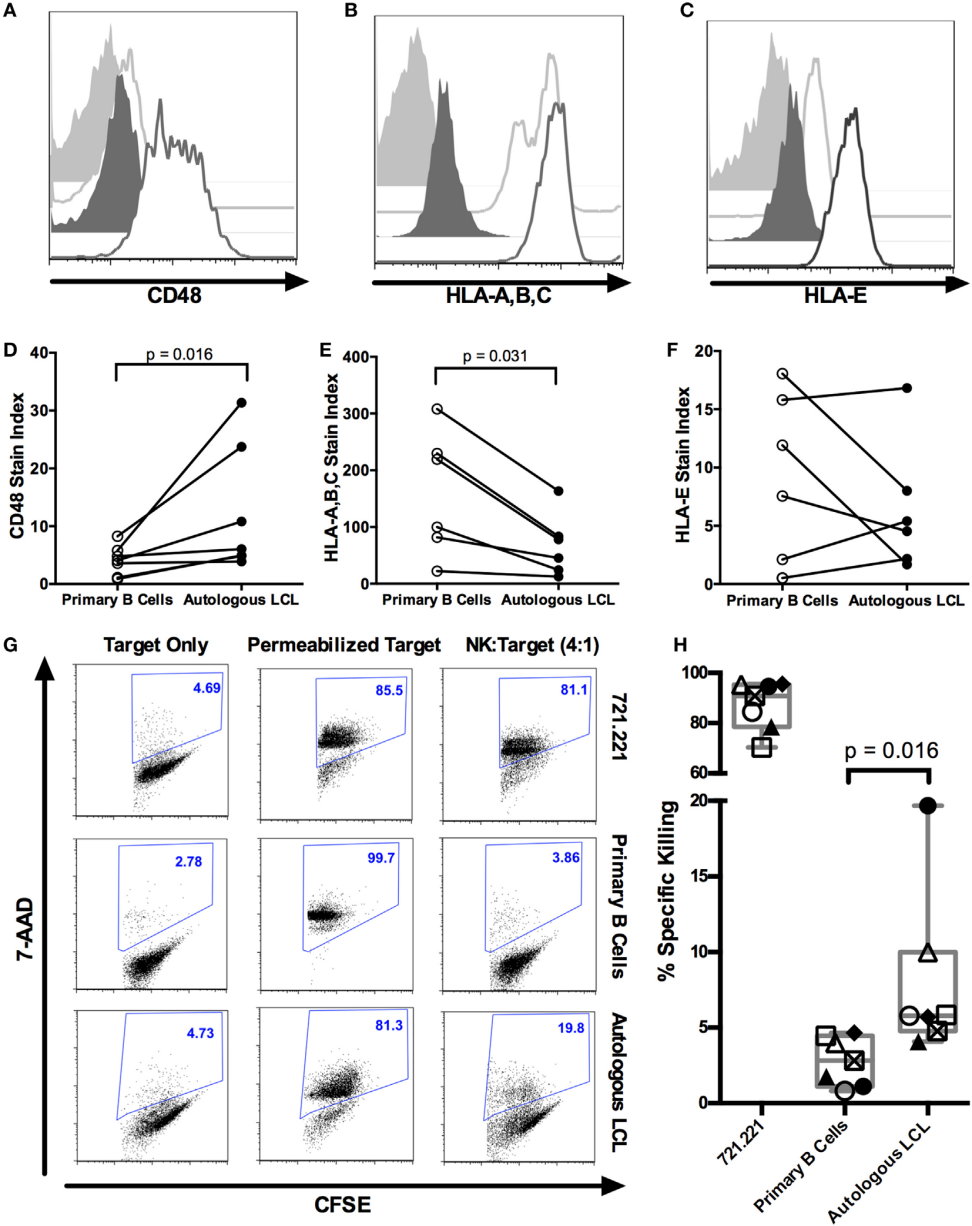
**NK cells kill autologous EBV^+^ LCL**. **(A–F)** Expression of NK ligands CD48, HLA-A,B,C, and HLA-E on autologous CD19^+^ B cells and EBV^+^ LCL. **(A–C)** Representative flow cytometry shows isotype (light gray shaded) or NK ligand-stained (light gray outline) primary CD19^+^ B cells or isotype (dark gray shaded) or NK ligand-stained (dark gray outline) autologous LCLs. **(D–F)** Stain indices (34) of *N* = 6 donors. **(G)** NK killing of CFSE-labeled target cells (721.221, primary B cells and autologous LCL target cells). The percentage of dead target cells, as detected by 7-AAD and CFSE, are shown in representative samples. **(H)** Specific killing was calculated as described in Section “Materials and Methods.” Each of the *N* = 7 donors is labeled with a unique symbol. Error bars represent the minimum and maximum values for each condition. All *p*-values were calculated using the Wilcoxon matched-pairs signed rank test.

Lymphoblastoid cell lines are CD19^+^ EBV-transformed B cell lines which display a viral latency type III pattern of gene expression and resemble EBV-LPDs like posttransplant lymphoproliferative disorder (35). We, therefore, analyzed expression of NKR ligands on LCL to evaluate how these cells, as a model of predominantly latent infection, stimulate NK cell recognition. We compared NK cell ligand staining on EBV^+^ LCL with primary autologous CD19^+^ B cells (Figure 1; Figure S1A in Supplementary Material). To account for the increased autofluorescence of EBV^+^ LCL, we used the stain index (34) to normalize expression levels to the corresponding isotype staining (Figures 1A–F). After normalization, EBV^+^ LCL (*n* = 6) display increased expression of the 2B4 ligand CD48 [Figures 1A,D (36)], lower expression of the inhibitory ligand MHC class I (HLA-A,B,C; Figures 1B,E), and no significant difference in expression of the NKG2A and NKG2C ligand HLA-E (Figures 1C,F) compared to autologous, primary CD19^+^ B cells.

### NK Cells Kill Autologous EBV^+^ LCL

We next tested the ability of purified NK cells to kill autologous EBV^+^ LCL using a flow cytometry-based cytotoxicity assay (Figures S1B,C in Supplementary Material; Figure 1G). NK cells robustly kill the MHC-I^lo^ positive control cell line 721.221 target (87.1 ± 3.7% SEM, Figure 1H). NK cells from healthy donors (*n* = 7) display significantly more cytotoxicity against autologous EBV^+^ LCL than against primary unstimulated B cells (8 ± 2.1% SEM versus 2.8 ± 0.6% SEM, *p* = 0.016; Figure 1H). The NK cytotoxicity against autologous EBV^+^ LCL is likely predominantly directed at latently infected LCL, because less than 2% of EBV^+^ LCL express the lytic antigen BZLF1 (Figure S3 in Supplementary Material) (6). Thus, transformed B cells harboring a latent EBV infection can elicit a cytotoxic response by autologous NK cells.

**Figure 2:**
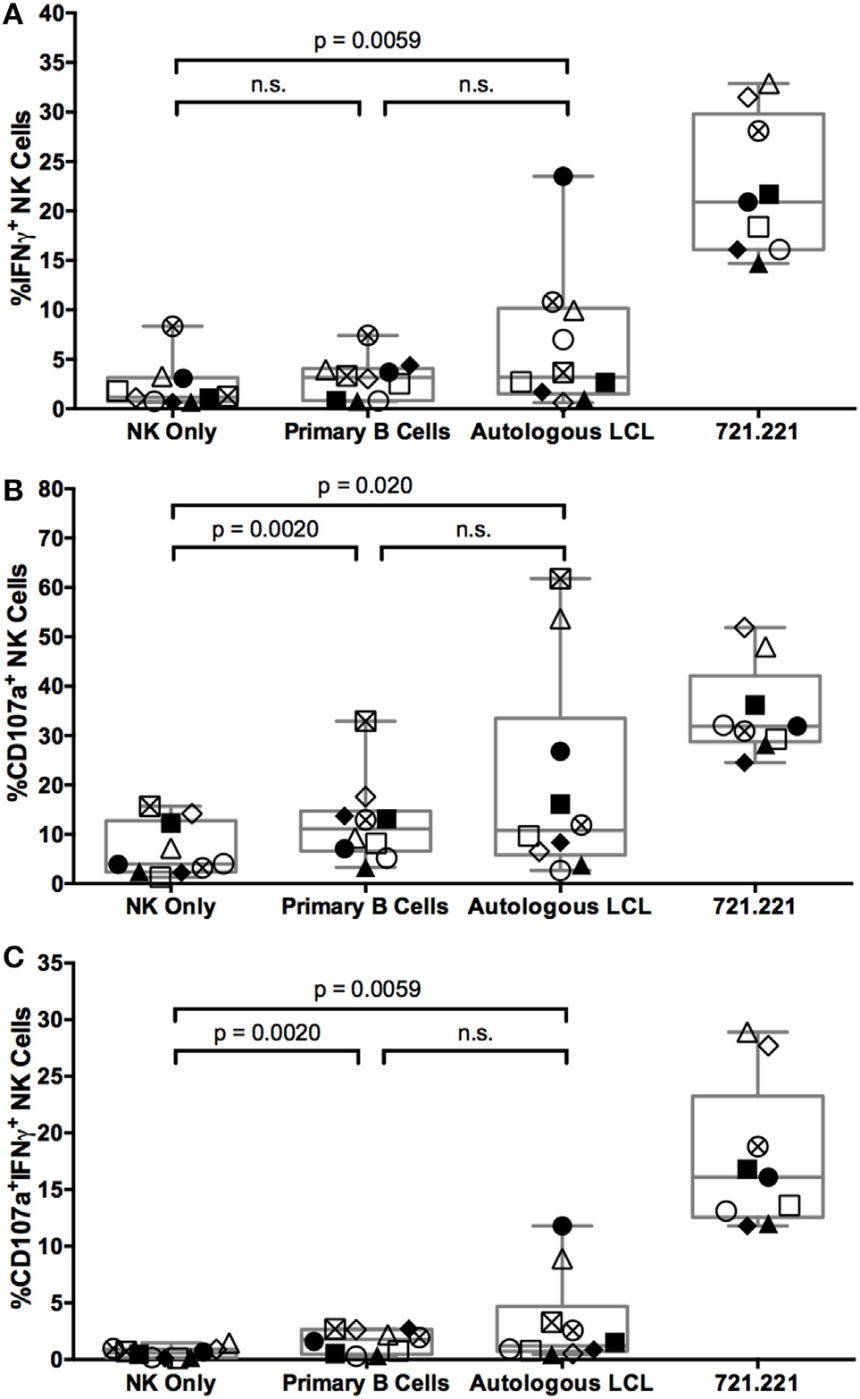
**NK cells respond to autologous LCL and primary B cells with similar frequency**. IFNγ **(A)**, CD107a **(B)**, or both **(C)** expression on NK cells (CD3^−^CD14^−^CD19^−^CD56^+^) alone (NK only) or after coculture with target cells (721.221 cells, primary B cells, or autologous LCLs). Error bars represent the minimum and maximum values for each condition. Each of the *N* = 10 donors is labeled with a unique symbol. All *p*-values were calculated using the Wilcoxon matched-pairs signed rank test.

To evaluate the NK cell activity during the targeting of autologous EBV-infected LCL, cocultures were stained with a panel of antibodies to identify NK cell subsets and their function (Figure S2A in Supplementary Material). NK cells (defined as CD3^+^CD14^+^CD19^+^CD56^+^ live singlets, Figure S2B in Supplementary Material) capable of releasing cytotoxic granules and/or producing cytokines were identified by staining for CD107a mobilization and intracellular IFNγ, respectively. In response to the positive control cell line 721.221, 20 ± 2% SEM of NK cells produce IFNγ, 29 ± 2% SEM of NK cells degranulate, and 17 ± 2% SEM of NK cells both degranulate and produce IFNγ above NK only background (Figure 2). In response to the autologous EBV^+^ LCL, 4.1 ± 2% SEM of NK cells produce IFNγ, 13.5 ± 6% SEM degranulate, and 2.6 ± 1.2% SEM of NK cells both degranulate and produce IFNγ above NK only background levels in NK only conditions (Figure 2). Thus, a small population of NK cells can respond to autologous EBV^+^ LCL.

### NKG2A Expression Defines a Subset of NK Cells Enriched in the Ability to Respond to Autologous EBV^+^ LCL

**Figure 3:**
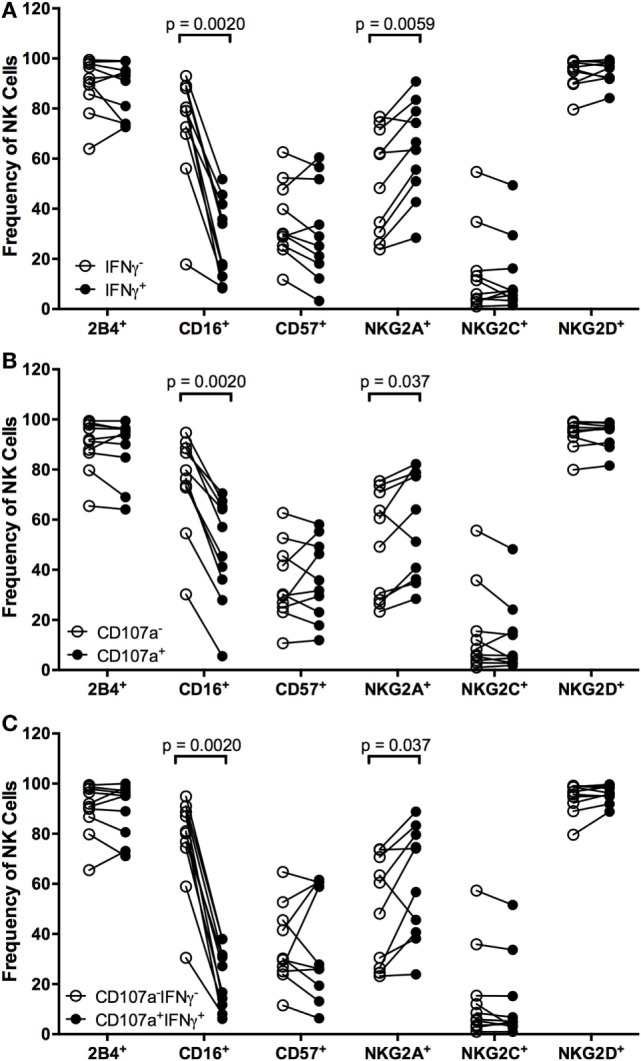
**Higher frequencies of NK cells responding to autologous LCL are NKG2A^+^**. Frequency of 2B4^+^, CD16^+^, CD57^+^, NKG2A^+^, NKG2C^+^, and NKG2D^+^ cells within IFNγ^−^ or IFNγ^+^ **(A)**, CD107a^−^ or CD107a^+^ **(B)**, and CD107a^−^IFNγ^+^ or CD107a^+^IFNγ^+^ **(C)** NK populations after coculture with autologous LCL (*N* = 10). All *p*-values were calculated using the Wilcoxon matched-pairs signed rank test.

To identify markers that can distinguish NK cells that respond to EBV^+^ LCL, we examined expression of the activating receptors CD16, 2B4, NKG2C, and NKG2D, the inhibitory receptor NKG2A, and the maturity marker CD57 on NK cells that had produced IFNγ and/or degranulated after coculture with autologous EBV^+^ LCL. Within an individual, each marker is expressed on a subset of total NK cells (Figure S4A in Supplementary Material). Expression of NK cell markers also varied among individuals. For example, the frequency of NKG2A^+^ cells in the total NK population ranges from 23.6–76.3%, in line with other published data (19, 37). Responding (IFNγ^+^ and/or CD107a^+^) NK cells are far less likely to express CD16 than non-responding (IFNγ^+^ and/or CD107a^−^) NK cells (Figure 3), consistent with the fact that CD16 expression is downregulated upon NK cell activation (38, 39). There are no significant differences in the frequencies of responding versus non-responding NK cells that were 2B4^+^, CD57^+^, NKG2C^+^, or NKG2D^+^ (Figure 3). In contrast, the frequency of NK cells mounting a functional response to autologous EBV^+^ LCL is significantly enriched for expression of NKG2A compared to NK cells that do not mount a functional response (Figure 3).

**Figure 4:**
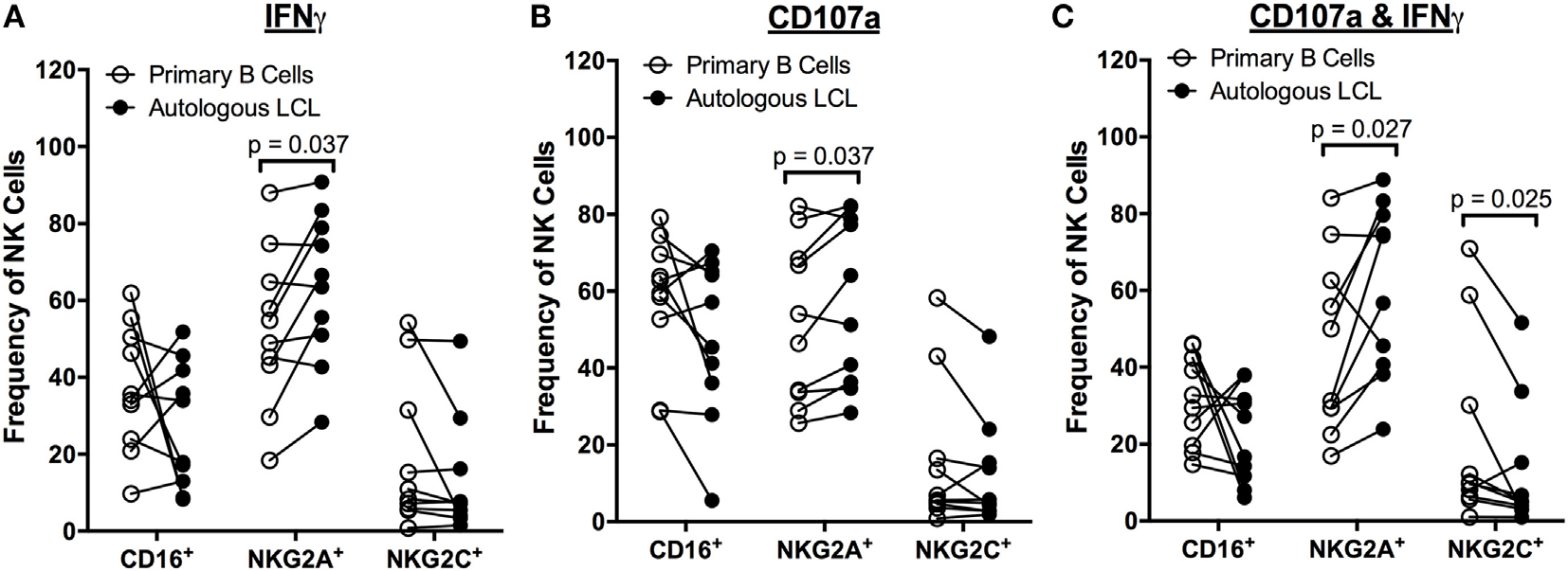
**Higher frequencies of NK cells respond specifically to autologous LCL and not primary B cells are NKG2A^+^**. Frequency of CD16^+^, NKG2A^+^, and NKG2C^+^ cells within IFNγ^+^ **(A)**, CD107a^+^ **(B)**, and CD107a^+^IFNγ^+^ **(C)** NK populations after coculture with primary B cells (open circles) or autologous LCL (filled circles) from *N* = 10 donors. All *p*-values were calculated using the Wilcoxon matched-pairs signed rank test.

To determine whether the population of NK cells that respond to EBV^+^ LCL is distinct from those that respond to uninfected B cells, we compared the expression of the markers CD16, CD57, 2B4, NKG2A, NKG2C, and NKG2D on NK cells responding to autologous EBV^+^ LCL versus primary B cells. There are no significant differences in the frequency of NK cells expressing CD16 in NK cells responding to primary B cells versus autologous EBV^+^ LCL (Figure 4). In contrast, the population of NK cells responding to autologous, EBV^+^ LCLs contains significantly higher frequencies of NKG2A^+^ cells than the NK cells responding to primary B cells (Figure 4). Moreover, the proportion of NKG2C^+^ cells in the subset of CD107a^+^IFNγ^+^ NK cells that respond to autologous EBV^+^ LCL is reduced (Figure 4C). There are no significant differences in the percentage of 2B4^+^, CD57^+^, or NKG2D^+^ NK cells responding to autologous EBV^+^ LCLs versus primary B cells (Figure S4 in Supplementary Material). Together, these data suggest that expression of NKG2A can distinguish a subset of NK cells that can specifically respond to B cells displaying latent EBV infection.

**Figure 5:**
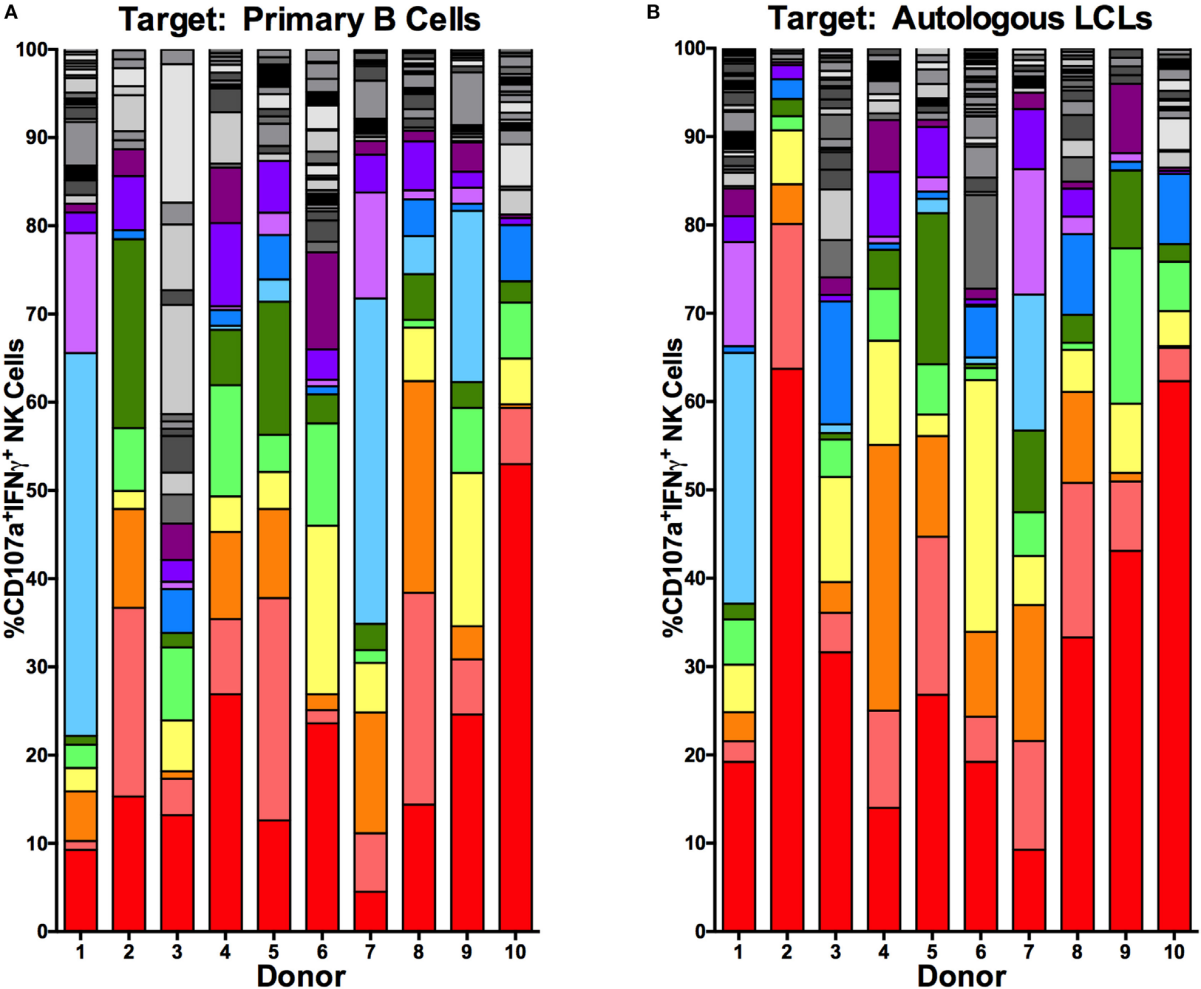

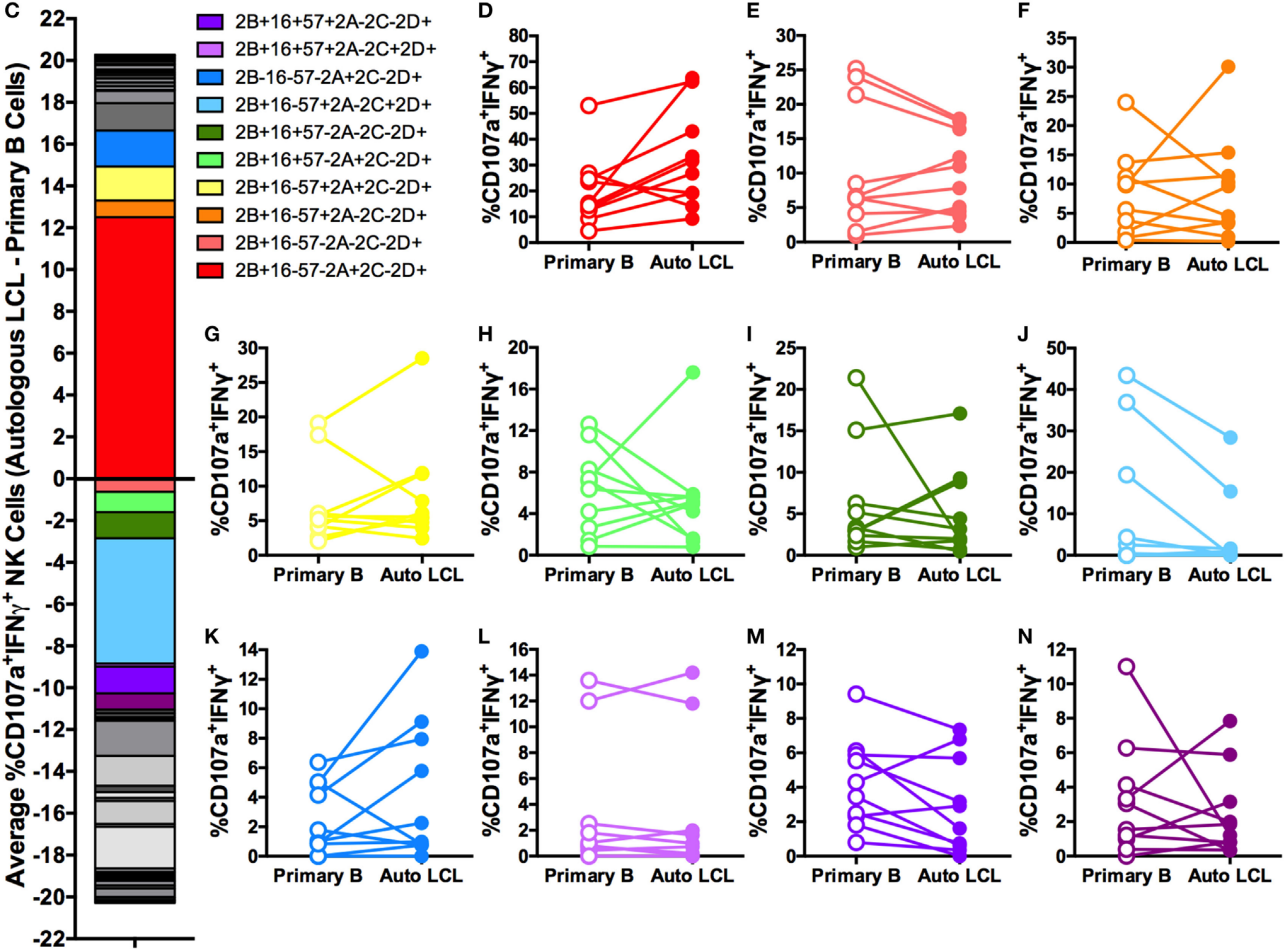
**Higher frequencies of NK cells responding specifically to autologous LCL are NKG2A^+^2B4^+^CD16^−^CD57^−^NKG2C^−^NKG2D^+^**. Visualization of the frequencies of 64 different NK receptor combinations in each of *N* = 10 donors within CD107a^+^IFNγ^+^ NK populations cocultured with primary B cells **(A)** and autologous LCL **(B)**. The 11 most frequent receptor combinations are shown in color, and the legend describes these combinations of 2B4 (2B), CD16 (16), CD57 (57), NKG2A (2A), NKG2C (2C), and NKG2D (2D). **(C)** The average difference in each receptor combination between CD107a^+^IFNγ^+^ NK cells from cocultures with primary B cells or autologous LCLs and **(D–N)** comparisons of the frequencies between CD107a^+^IFNγ^+^ NK populations from coculture with primary B cells (open circles) and autologous LCL (filled circles). All *p*-values were calculated using the Wilcoxon matched-pairs signed rank test. A Bonferroni correction was used to adjust for multiple comparisons.

To further characterize the NK cell subsets responding to latent EBV^+^ LCL, we examined the NK cell response to EBV using combinations of the six NKRs 2B4, CD16, CD57, NKG2A, NKG2C, and NKG2D. Using a Boolean approach, we determined the frequency of each of the 64 potential NK cell subsets defined by expression of the six NK cell receptors on NK cells responding, on the basis of CD107a and/or IFNγ expression, to coculture with primary B cells or autologous LCL (shown for each donor in Figures 5A,B, respectively). To identify EBV-responsive NK cell subsets, we normalized the frequency of NK cells responding to autologous LCL to the frequency responding to primary B cells. The average normalized percentage of each of these subsets among the 10 donors is displayed in Figure 5C. This analysis suggests that the NKG2A^+^2B4^+^CD16^−^CD57^−^NKG2C^−^NKG2D^+^ NK cell population is the most responsive against EBV-infected LCL, as it was consistently more responsive to autologous LCL than to primary B cells, though this effect was not statistically significant after controlling for multiple comparisons (Figures 5D–N). Consistent with these observations, the diversity of the responding cells (CD107a^+^IFNγ^+^) was lower in 8 of 10 donors cocultured with autologous LCL compared to primary B cells (Figure S5 in Supplementary Material); this reduced diversity also suggests that the specific response to EBV infection may be driven by a subset of EBV-responsive NK cells. Together, these data suggest that a subset of NK cells, identified as NKG2A^+^2B4^+^CD16^−^CD57^−^NKG2C^−^NKG2D^+^, can respond specifically to EBV^+^ LCL.

**Figure 6:**
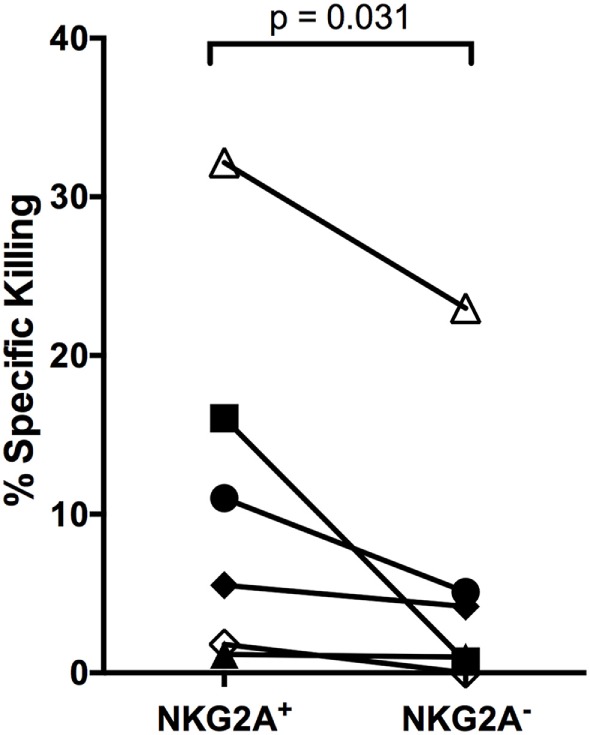
**NKG2A^+^ NK cells kill autologous LCL better than NKG2A^−^ NK cells**. NKG2A^+^ and NKG2A^−^ NK cells were sorted and placed in a killing assay with CellTrace Violet-labeled autologous LCL targets at a 4:1 effector:target ratio. Specific killing was calculated as described in Section “Materials and Methods.” Each of the *N* = 6 donors is labeled with a unique symbol. All *p*-values were calculated using the Wilcoxon matched-pairs signed rank test.

To better define the NK cell subset responding to autologous LCL, we evaluated NK cell IFNγ secretion and CD107a activity in the presence of antibodies that were previously reported to block NKG2D, NKG2A, and 2B4. Blocking NKG2D led to a consistent decrease in the frequency of NK cells producing IFNγ and CD107a, but the effects of NKG2A and 2B4 blocking were less dramatic, in some cases enhancing activity (Figure S6 in Supplementary Material). This implies that NKG2D is involved in the recognition of autologous LCL, but cannot explain the entire effect. As NKG2A is an inhibitory receptor, it is likely that it may not be directly involved in killing but instead may mark a responsive subset. Thus, to confirm the role of NKG2A-expressing NK cells in the response to autologous EBV^+^ LCL, we compared the ability of NKG2A^+^ and NKG2A^−^ NK cells from the same donor for their ability to kill autologous EBV^+^ LCL. In all six donors, NKG2A^+^ NK cells display higher killing than did NKG2A^−^ NK cells (Figure 6; Figure S7 in Supplementary Material, *p* = 0.03), confirming that NK cell subsets that are functionally responsive to EBV^+^ LCL are enriched for NKG2A expression.

## Discussion

Latent infection dominates the EBV life cycle, and failure to adequately control latent EBV infection has serious consequences including the development of a spectrum of EBV-LPD. NK cells represent previously under-appreciated players in the immune response to EBV infection. Here, we demonstrate that NK cells display cytotoxicity specifically against autologous EBV^+^ LCL. Compared to host B cells, autologous EBV^+^ LCL upregulate expression of the NK activating ligand CD48 and reduce expression of the NK inhibitory ligand MHC class I, potentially making them targets of NK cell-mediated lysis. Consistent with this observation, we find that NKG2A-expressing NK cells are particularly enriched for the ability to respond to autologous EBV^+^ LCL. Thus, while prior studies highlighted the ability of NK cells to distinguish active, lytic infection (4, 7, 27), our data indicate that NK cells are capable of distinguishing autologous EBV^+^ LCL from healthy B cells, potentially laying the groundwork for harnessing their activity in the treatment of EBV-LPD.

The majority of studies on the NK cell response to EBV have focused on NK cells during primary infection or upon lytic reactivation of the virus. NK cell activity has been shown to be greater against lytically infected targets than against the corresponding latently infected targets (6, 7). However, these studies used the allogeneic Akata target cell lines, raising the possibility that “missing self” targeting by NK cells could have contributed to the activity observed. Our data demonstrating that NKG2A-expressing NK cells predominate in the NK response to latently infected LCL represents a clinically relevant autologous interaction given that latency is the major phase of the EBV life cycle. Our analysis takes into account any relevant non-specific activity against uninfected host B cells, allowing us to identify the subset of NK cells enriched in their responsiveness against the autologous EBV^+^ LCL. Further, we directly examine killing of target cells, rather than only NK-specific outcomes such as degranulation and IFNγ production as indirect measures for effects on target cells. Thus, despite the fact that the frequency of “responding” NK cells, as defined by expression of IFNγ or CD107a, was similar in cocultures with autologous B cells and EBV^+^ autologous LCL, our data clearly demonstrate that there is enhanced killing of EBV^+^ LCL by autologous NK cells. These results are consistent with the idea that NK cells can serially kill multiple targets (40–42), and explain why it has been hard to detect specific targeting of EBV^+^ LCL in past studies. This small subset of “serial” killers will be difficult to identify from the non-specific activity noted in cultures. Thus, here, we have phenotypically identified the small frequency of NK cells that account for the specific targeting of LCL.

We demonstrate that NKG2A-expressing NK cells are enriched for activity against latent EBV-infected LCL, with a more granular definition of NKG2A^+^2B4^+^CD16^−^CD57^−^NKG2C^−^NKG2D^+^ NK cells. Further, blocking experiments suggest that NKG2D may play a role in the recognition of autologous LCL, but likely other receptors are involved. The absence of CD57 expression and the presence of NKG2A expression suggests that these are relatively immature NK cells since terminally differentiated NK cells typically lose expression of NKG2A and gain expression of CD57 (21, 43). NKG2A^+^CD57^−^ NK cells have also been identified as important during other stages of EBV infection and thus may be relevant in innate regulation of EBV throughout its life cycle. EBV-responsive NK cells from the peripheral blood during primary symptomatic EBV infection have been described as CD56^dim^KIR^−^NKG2A^+^ (7), while those persisting after acute EBV infection are CD56^dim^NKG2A^+^CD57^+^ (28). Within the tonsils, an IFNγ^hi^CD56^bright^NKG2A^+^CD94^+^CD54^+^CD62L^−^ NK cell subset has been implicated as the EBV-responsive NK cell that restricts primary B cell infection (27). In fact, the age-related decline in immature NK cells (CD56^dim^NKG2A^+^NKR^−^NKG2C^−^CD57^−^), which make up the predominant subset in newborn infants and humanized mice, may reduce NK control of EBV infection and predispose adolescents for EBV^+^ IM (44). Thus, multiple lines of evidence suggest that NKG2A^+^CD57^−^ NK cells play a critical role in controlling EBV infection. It will, therefore, be of interest to study if the relative abundance of NKG2A^+^CD57^−^ NK cells predicts susceptibility to EBV-LPD or EBV^+^ IM, or if these subsets can be expanded *in vivo* or *ex vivo* and used therapeutically.

Common among all studies of the NK cell response to EBV is the involvement of NKG2A, an NK inhibitory receptor, as a marker of EBV-responsive NK cells. The role of the inhibitory receptor NKG2A in NK cell responses to EBV remains unclear. NKG2A^+^ NK cells are also enriched in the ability to respond to HIV (45). This observation is explained by the presentation of a conserved HIV peptide by HLA-E that abrogates the interaction between NKG2A and HLA-E, resulting in the loss of this inhibitory signal and sensitization to killing by NKG2A^+^ NK cells (45). A similar mechanism could play a role in EBV, particularly since HLA-E is not downregulated by either HIV or EBV (46). It will be of interest to determine if EBV similarly expresses a conserved peptide that allows escape from NK cell inhibition mediated by the interaction of NKG2A and HLA-E. Alternately, it is possible that NKG2A is a marker of the EBV-responsive NK cell population but plays no direct role in NK cell function against EBV-infected cells. Most NKG2A-expressing NK cells do not co-express KIR (47–49), so these data could suggest that the absence of the inhibitory KIR receptors may allow this subset to be more activate against autologous LCL. Similarly, our data suggest that NGK2D may play a role in recognition of autologous LCL, since blocking NKG2D reduces NK cell IFNγ secretion by approximately 30%, strongly suggesting that multiple receptors are involved in the recognition of autologous LCL.

Our data suggest that the response to latent EBV infection may result from a shift in the balance from NK inhibition toward NK activation. Specifically, we observed reduced expression of the NK inhibitory ligands MHC class I and increased expression of the NK-activating ligand CD48 on EBV^+^ LCLs compared to host B cells. While Azzi et al. (7) reported an increase in MHC class I expression on autologous LCLs compared to CD19^+^ B cells from PBMCs, it is unclear if they accounted for differences in autofluorescence between the two cell types. In accord with our results, LCLs generated from an EBV strain lacking the latent protein LMP2a display increased MHC class I expression, suggesting that latent viral genes can downregulate MHC class I expression (50). Along similar lines, the downregulation of inhibitory ligands and an upregulation of activating ligands are thought to contribute to the NK response toward lytic EBV. In fact, the further downregulation of MHC class I during lytic infection and the upregulation of additional activating ligands, including CD112, ULBP1, MICA, and CD155 (6, 7), may help explain the difference in magnitude of the NK response to lytically versus latently EBV-infected B cells. Ultimately, all NK studies are limited by our knowledge of verified ligands for NK cell receptors. Further studies enumerating the differential regulation of NK ligand expression between lytic and latent EBV infection in B cells may provide additional mechanistic insight into NK cell control of EBV infection.

Our results provide the first clear demonstration of a significant NK cell response to autologous EBV^+^ LCL as a model of latent EBV infection. These data help to complete our understanding of the role of NK cells throughout the EBV life cycle and may lay the groundwork for developing novel NK-based therapeutics for EBV-LPD.

## Ethics Statement

This study was carried out in accordance with the recommendations of the International Compilation of Human Research Standards from the Office of Human Research Protections at the US Department of Health & Human Services with written informed consent from all subjects. All subjects gave written informed consent in accordance with the Declaration of Helsinki. The protocol was approved by the Stanford University Institutional Review Board.

## Author Contributions

OH, DS-A, NZ, SK, OM, and CB conceived and designed the research; OH, MH, JP, and NZ performed the research; OH, DS-A, NZ, SK, OM, and CB analyzed data; and OH, DS-A, SK, OM, and CB wrote the paper. All the authors agree to be accountable for the content of the work.

## Conflict of Interest Statement

The authors declare that the research was conducted in the absence of any commercial or financial relationships that could be construed as a potential conflict of interest.
