# Supplementary material for: NKG2A-Expressing Natural Killer Cells Dominate the Response to Autologous Lymphoblastoid Cells Infected with Epstein–Barr Virus
Source: Front Immunol. 2016 Dec 15;7:607. doi: 10.3389/fimmu.2016.00607 (PMC5156658; doi:10.3389/fimmu.2016.00607)
Supplement: Supplementary file 1 [file Data_Sheet_1.PDF]

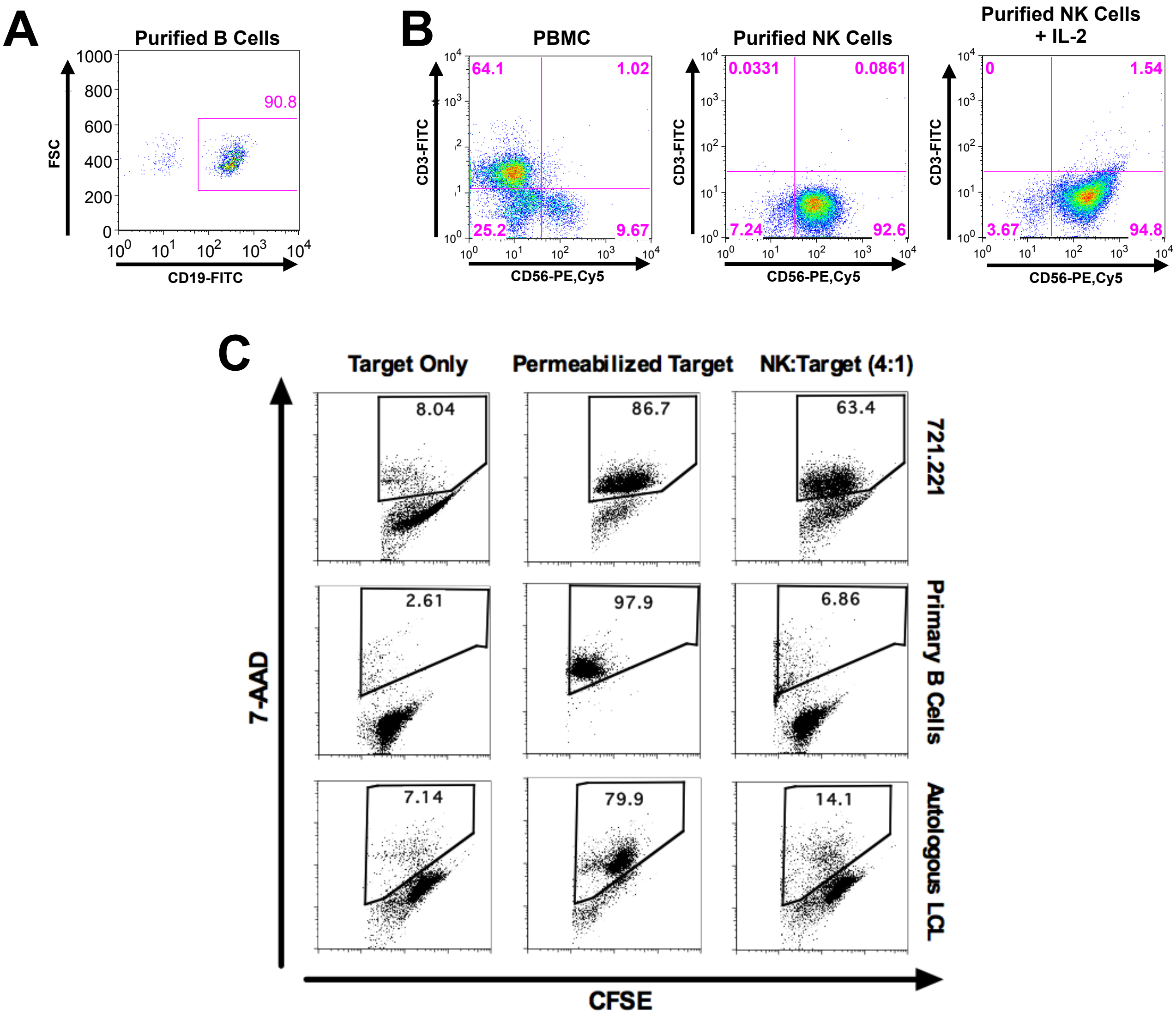

Supplemental Figure 1. Primary Cell Isolation and NK Killing Assay. (A-B) Primary human NK cells and B cells were isolated from healthy donors using the RosetteSep Human B Cell (A) and NK Cell (B) enrichment kits. To assess purity, samples were then stained for CD19 (A) or CD3 and CD56 (B) to determine purity of NK or B cell isolations, respectively. (A) Representative plot displaying the frequency of purified $CD19^{+}$ B cells. (B) Representative plot from a single donor displaying the frequency of NK cells ($CD3^{-}CD56^{+}$) in PBMC, purified NK cells, and purified NK cells after 2 days culture with 300 U/mL IL-2. (C) NK killing of CFSE-labeled target cells (721.221, primary B cells, and autologous LCL target cells). The percentage of dead target cells, as detected by 7-AAD and CFSE, are shown in representative samples.

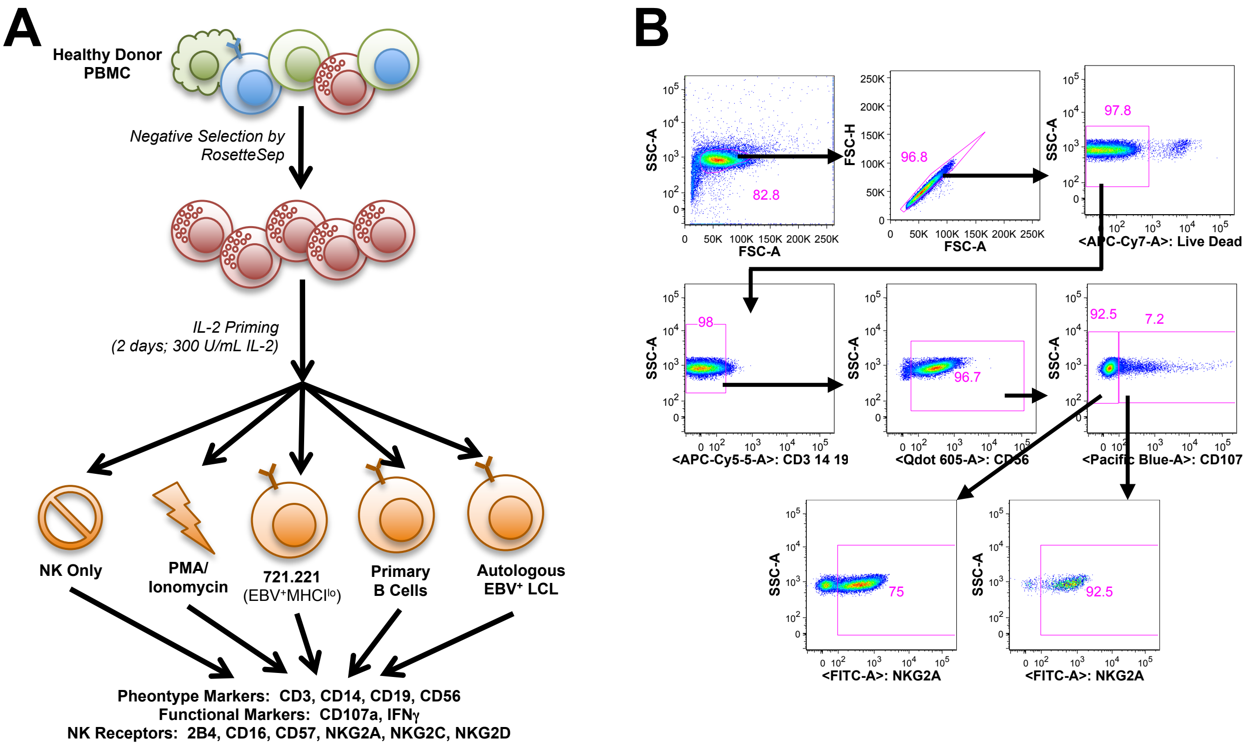

Supplemental Figure 2. Co-Culture and Gating Strategy. (A) Co-culture strategy. Primary human NK cells were negatively selected from healthy donor samples using the RosetteSep Human NK cell enrichment kit and subsequently cultures for 2 days in 300 U/mL IL-2. IL-2 primed NK cells were then cultured under the following conditions: (1) NKs only, (2) treated with PMA/Ionomycin, (3) 1:1 NK : 721.221, (4) 1:1 NK : primary B, (5) 1:1 NK : autologous LCL. After 4 hours, co-cultures were assayed by flow cytometry for 2B4, CD3, CD14, CD16, CD19, CD107a, CD56, CD57, IFNγ, NKG2A, NKG2C, NKG2D, and a Near-IR Live-Dead stain. (B) Example gating strategy. Cells were first gated on forward and side-scatter, followed by gating for singlets and live cells. B cells, T cells, and monocytes were excluded from analysis by gating for $CD3^{-}CD14^{-}CD19^{-}$ cells. Cells were then gated for expression of the NK cell marker CD56. All $CD56^{+}$ NK cells were then analyzed for functionality. Within the functionally responding ($CD107a^{+}$ or $IFN\gamma^{+}$) or non-responding ($CD107a^{-}$, $IFN\gamma^{-}$), expression of NK cell receptors, like NKG2A, was analyzed. All gates were set on fluorescence minus one (FMO)-stained PBMCs.

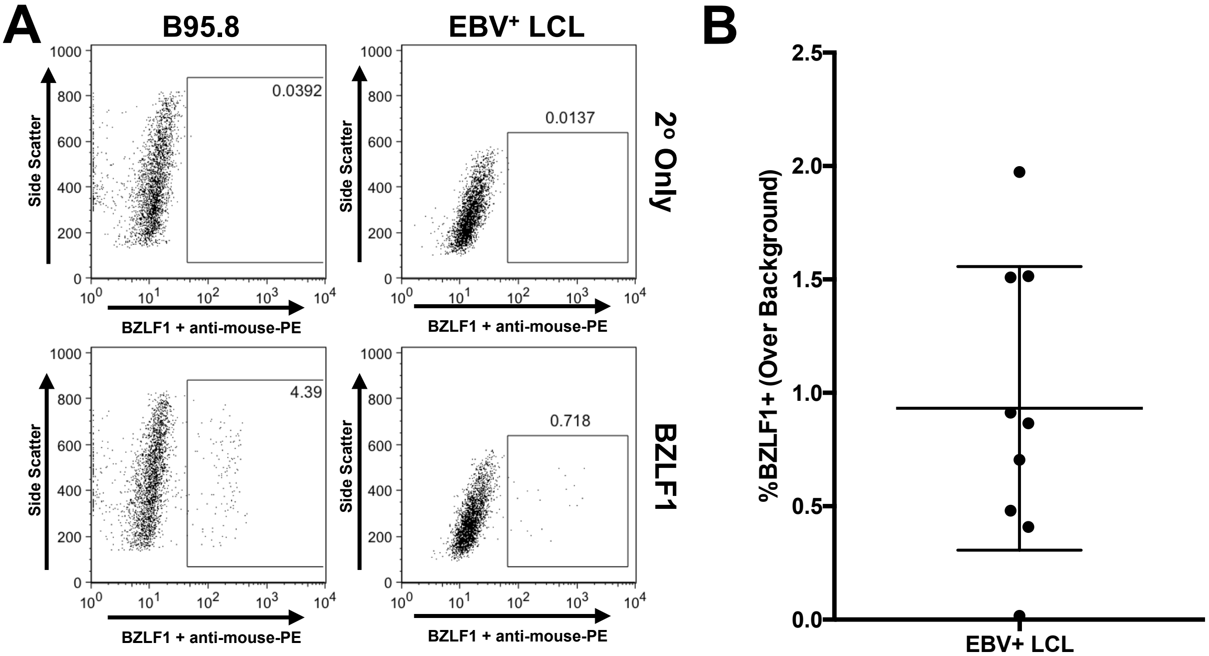

Supplemental Figure 3. BZLF1 Expression in EBV$^+$ LCL. The lytically-infected EBV$^+$ B95.8 marmoset line and EBV+ LCL were stained with BZLF1 and a PE-conjugated secondary antibody or secondary antibody alone as a control. (A) Representative staining of BZLF1 in the lytically-infected EBV$^+$ B95.8 marmoset line and a representative EBV$^+$ LCL. (B) The proportion of BZLF1$^+$ cells in EBV$^+$ LCLs, after background (2° only) is subtracted.

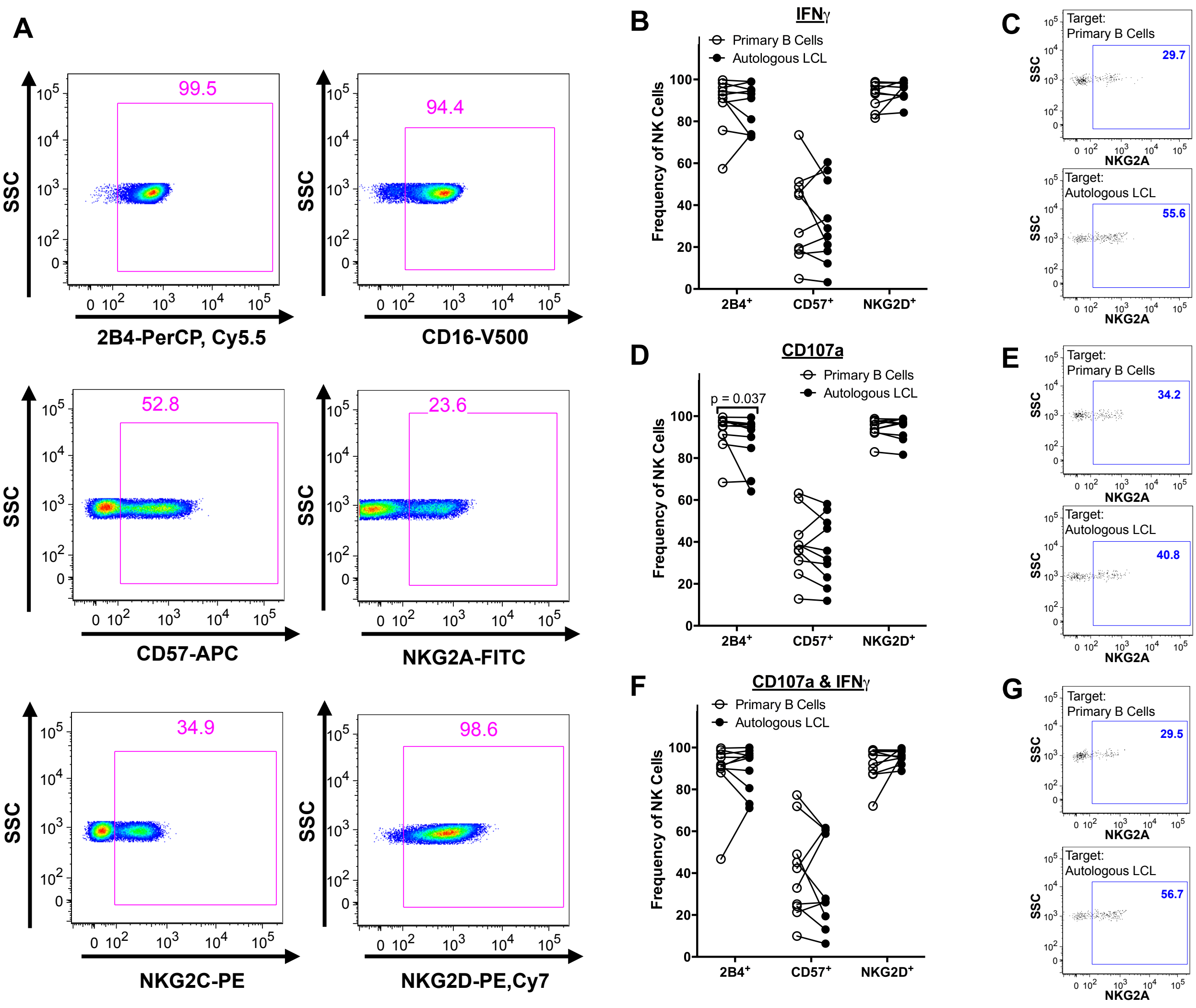

Supplemental Figure 4. Individual Receptor Analysis & Example Flow Plots. (A) Representative stains for 2B4, CD16, CD57, NKG2A, NKG2C, and NKG2D on $CD3^-CD14^-CD19^-CD56^+$ NK cells. (B-G) Frequency of 2B4, CD57, and NKG2D on $IFN\gamma^+$ (B), $CD107a^+$ (D), and $CD107a^+IFN\gamma^+$ (F) NK cells after co-culture with primary B cells or autologous LCL from N=10 donors. Representative plots showing NKG2A expression in $IFN\gamma^+$ (C), $CD107a^+$ (E), or $CD107a^+IFN\gamma^+$ (G) NK cells after co-culture with primary B cell or autologous LCL. All p-values were calculated using the Wilcoxon matched-pairs signed rank test.

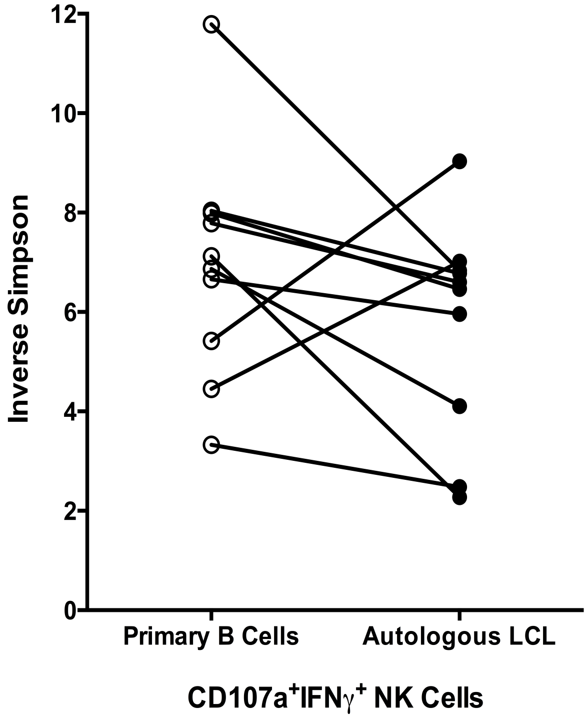

Supplemental Figure 5. Diversity Analysis of NK Cell Populations. Inverse Simpson analysis for the all CD107a^{+}IFNγ^{+} NK cells, comparing diversity of NK cells co-cultured with primary B cells versus autologous LCL.

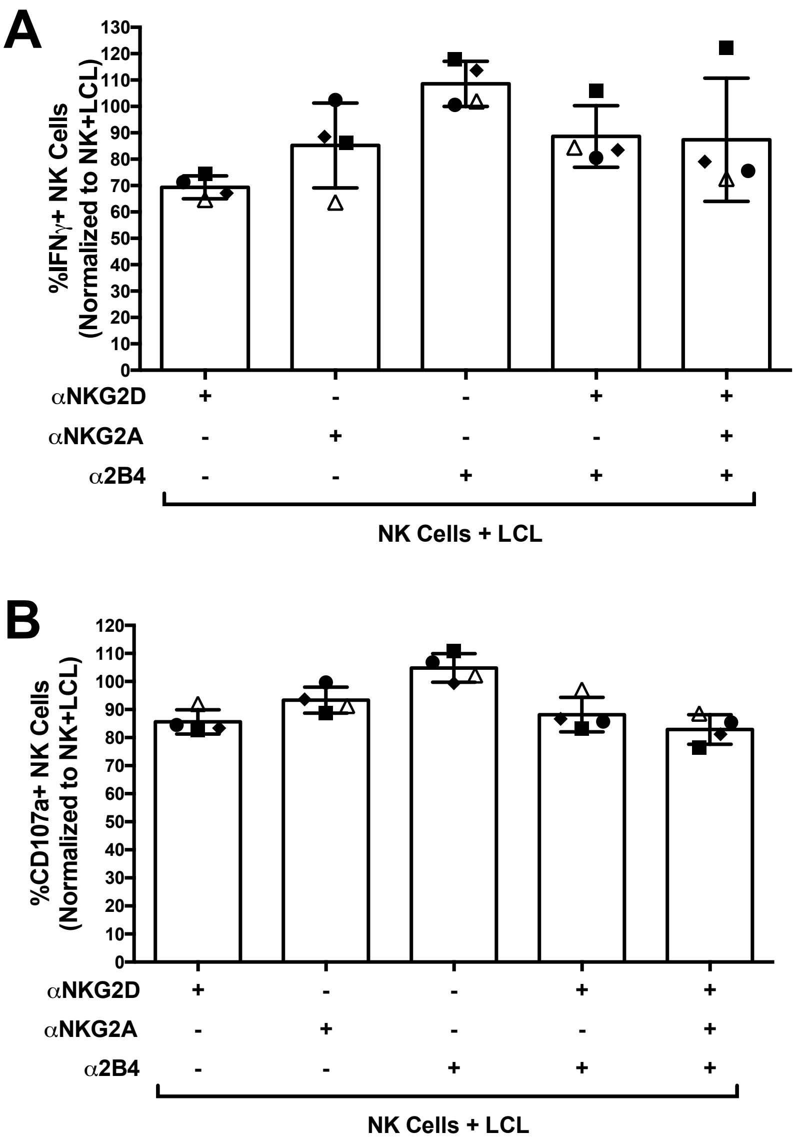

Supplemental Figure 6. Receptor Blocking and NK Functional Responses to LCL. PBMCs were isolated from whole blood with Ficoll-Paque (GE Healthcare), and NK cells purified by negative isolation (Miltenyi). NK cells were cultured in RPMI complete with 300U/ml IL-2 for 2 days. 2.5*10^5 NK cells were pre-blocked for 1h at 37C with anti-NKG2D (clone 1D11, Biolegend), anti-NKG2A (clone 131411, R&D) or anti-2B4 (clone C1.7, Biolegend), prior to adding 2.5*10^5 LCL target cells for a 1:1 E:T ratio, with a final blocking antibody concentration of 10ug/ml. Cells were cultured in RPMI complete with 300U/ml IL-2, 1x Brefeldin A and Monensin (eBioscience), and anti-CD107a-APC H7 (BD Pharmingen). At the end of the co-culture, cells were stained with LIVE/DEAD Fixable Yellow Dead Cell Stain Kit (Molecular Probes), washed, and then stained with anti-CD3-PE (Biolegend), anti-CD19-APC (Biolegend), anti-CD20-FITC (Biolegend) and anti-CD56-PE Cy7 (BD Pharmingen). Cells were fixed and permeabilized with FACS Lyse and FACS Perm II (BD Pharmingen) and stained with anti-IFNγ-V450 (BD Pharmingen), and run on a MACSQuant Analyzer.

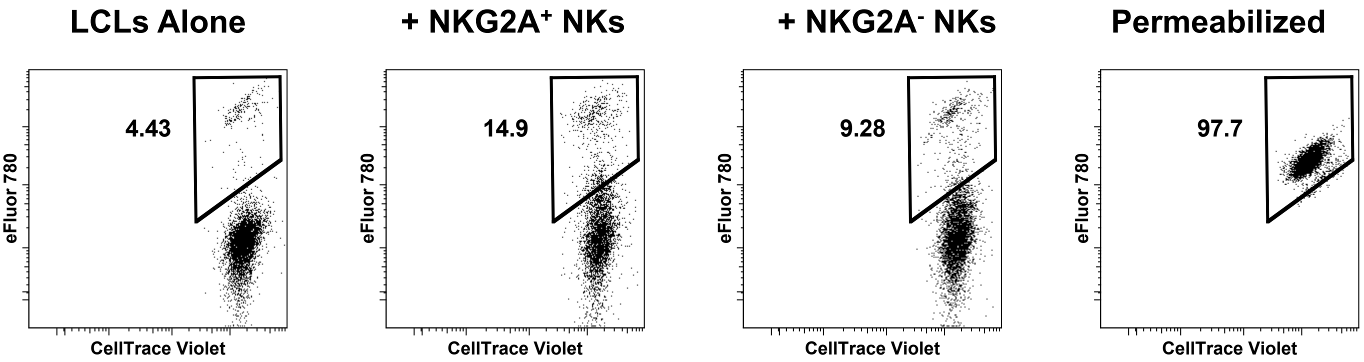

Supplemental Figure 7. NKG2A$^+$ NK cells kill autologous LCL better than NKG2A$^-$ NK cells. NKG2A$^+$ and NKG2A$^-$ NK cells were sorted and placed in a killing assay with CellTrace Violet-labeled autologous LCL targets at a 4:1 Effector:Target ratio. Flow plots from a representative donor are shown.
